# Assessing Dietary Intake Patterns Through Cluster Analysis Among Adolescents in Selected Districts of Bihar and Assam From India: A Cross-Sectional Survey

**DOI:** 10.3389/fnut.2021.592581

**Published:** 2021-11-25

**Authors:** Shantanu Sharma, Sonali Maheshwari, Jitesh Kuwatada, Chandrashekhar, Sunil Mehra

**Affiliations:** ^1^Department of Clinical Sciences, Lund University, Malmo, Sweden; ^2^Department of Reproductive, Maternal, Child, and Adolescent Health, MAMTA Health Institute for Mother and Child, New Delhi, India

**Keywords:** adolescent, diet surveys, food, meat, nutrition assessment, vegetables

## Abstract

**Background:** In the recent decade, dietary pattern assessment has evolved as a promising tool to describe the whole diet and represent inter-correlations between different dietary components. We aimed to derive the dietary patterns of adolescents (10–19 years) using cluster analysis on food groups and evaluate these patterns according to their socio-demographic profile.

**Methods:** This community-based cross-sectional study was conducted in two districts, each from Bihar and Assam in India. Adolescents (10–19 years) were enrolled from both rural and urban areas. The dietary intake was assessed through a pre-validated single food frequency questionnaire. Cluster analysis was performed by a 2-step procedure to explore dietary patterns, pre-fixed at 2 clusters. Clusters were analyzed with respect to socio-demographic characteristics using binomial logistic regression.

**Results:** A total of 826 girls and 811 boys were enrolled in the study. We found two major dietary patterns, namely a low- and high-mixed diet. The low-mixed diet (76.5% prevalence) had daily consumption of green vegetables, including leafy vegetables, with less frequent consumption of other foods. The high-mixed diet (23.5% prevalence) had more frequent consumption of chicken, meat, egg, and milk/curd apart from green vegetables. Adolescent boys had 3.6 times higher odds of consuming a low-mixed diet compared to girls. Similarly, adolescents with lower education grades and from marginalized social classes had two times higher odds of taking a low-mixed diet than their respective counterparts.

**Conclusions:** The high consumption of a low-mixed diet and relatively less milk consumption limit the comprehensive growth of adolescents. Improvement in dietary intake of adolescents from marginalized sections of society can prove to be an important deterrent in mitigating India's nutritional challenges.

## Introduction

Adolescence, defined by the World Health Organization as the age between 10 and 19, is a period of physical, cognitive, and psychosocial growth and development (1). It is estimated that men and women gain 50% of adult weight and 15% of adult height during adolescence. Hence, it is imperative to understand that adequate nutrition is important for attaining full growth potential, and any insults or poor nutrition during adolescence may have transgenerational consequences (2). Adolescence represents a window of opportunity to recuperate the growth insults that ensued early in life (3). There is a growing interest at the public health and policy levels in understanding the complex adolescent health and nutrition needs (1). Investment in adolescent health and well-being has been recognized as one of the best investments for achieving the United Nation's Sustainable Development Goals (4).

The world's 90% of adolescents are concentrated in low- and middle-income countries (5). India has the largest number of adolescents globally, ~243 million, and they have a huge potential to generate greater economic dividends for the nation (4). However, a large number of adolescents are vulnerable in India. For instance, 42 and 54% of adolescent girls aged 15–19 are thin (Body Mass Index (BMI) <18.5 Kg/m^2^) and anemic, respectively. Similarly, 45 and 29% of adolescent boys aged 15–19 are thin (BMI <18.5 Kg/m^2^) and anemic in India, respectively (6). According to the recent comprehensive national nutrition survey (2016–2018), 16–37% of adolescents had micronutrient deficiencies of different types, including deficiency of vitamin A, vitamin D, folate, zinc, and vitamin B12 (7).

The prevalence of undernutrition and micronutrient deficiencies is not equitable and differs across geographies, ethnicities, gender, religion, and other socio-demographic attributes (7). For example, adolescents in the early age group (10–14 years) and from marginalized families, rural areas, Hindu families, and the poorest of the poor socio-economic strata had the highest prevalence of vitamin A deficiencies compared to their respective counterparts. Similarly, adolescents with a vegetarian diet have been found to have high probabilities of micronutrient deficiencies (7). This spotlights the influence of various socio-demographic attributes in the nutrition of adolescents, and hence they need to be understood while comprehending the nutrition profile of the population.

For better understanding the nutrition profile of the population, it is imperative to consider the dietary patterns, argued to describe the whole of the diet better, and represent inter-correlations between different dietary components (3). Also, dietary patterns' assessment generates an evidence base and provides public health recommendations to interventionists (8). Many studies in the past have attempted to assess the dietary patterns of the Indian population (9–12). Most of these studies have identified a vegetarian dietary pattern based on fruits, vegetables, pulses, and cereals, with added dairy products, meat, and eggs in many cases. Large variations in the dietary patterns in different geographical regions have been noticed with higher consumption of fish or egg or meat in the Eastern parts compared to the Northern or Western parts of the country. However, it is crucial to note that adolescents have been underrepresented or not included in most of these studies (13).

Furthermore, most of the studies used principal component analysis (PCA) for finding patterns that may not be very useful in terms of interpretation (13). PCA does not represent the whole of the diet; instead, it describes patterns artificially composed of a few distinguishing food items without calculating the consumption of each food group in each pattern (14). On the contrary, cluster analysis groups individuals into distinct dietary patterns who share similar frequency patterns for food consumption. Besides, cluster analysis can be used for both categorical and continuous variables (15).

Considering the abovementioned gaps, we decided to explore dietary patterns among adolescents for developing a nutrition-specific intervention. The present study was a part of the formative assessment to inform the intervention aimed at improving the maternal, child, and adolescent health and nutrition conducted by MAMTA-Health Institute for Mother and Child, a not-for-profit institution. The project, entitled MANCH (Maternal, Adolescent, Newborn, and Child Health), was implemented across two districts, namely Munger and Darrang, each in Bihar and Assam, respectively. The specific objectives of the MANCH project were: (a) to improve the nutritional status of adolescents, pregnant women, and lactating mothers, (b) to improve access to public health services by them, and (c) to enhance self-efficacy of adolescents through life skills to prevent early marriage. The formative assessment was done at the beginning of the project to evaluate the baseline measures of the indicators corresponding to the project's objectives. However, the present study restricts itself to derive the dietary patterns of adolescents (10–19 years) using cluster analysis on different food groups and evaluate these patterns with respect to the selected socio-demographic profile.

## Methods

### Study Design

We conducted a community-based cross-sectional study of adolescents (girls and boys) in Bihar and Assam, situated in east and North-east part of India, respectively.

### Study Setting and Sampling

Situated to the east of the capital of Bihar, Munger has a population of 1.3 million, a sex ratio of 876 females per 1,000 males, 62% female literacy rate, and 31% of non-pregnant women (15–49 years) with undernutrition and 65% with anemia (6). Similarly, situated to the north of the capital of Assam, Darrang has a population of 0.93 million, a sex ratio of 935 females per 1,000 males, 58% of female literacy rate, and 27.5% of non-pregnant women (15–49 years) with undernutrition and 46% with anemia (6).

The states and districts were purposively selected as both were the part of a larger intervention project. The intervention areas comprised of 45 urban wards and 6 village-level local elected bodies called Gram Panchayats in Munger district of Bihar and; 4 Gram Panchayats in Darang district of Assam. The survey was concluded in 26 villages from 4 Gram panchayats in Darang (Ramhari, Chamuapara, Chapai, and Dahi). Similarly, survey was conducted in 52 villages from 6 Gram Panchayats of Munger (Shankarpur, Mahuli, Shreematpur, Kataria, Mayee, and Mirzapur Bardeh). Around 8 adolescent boys and 8 girls were selected per village or ward. One adolescent was selected per household, the selection of which is described below.

From the two selected districts, all the target villages and urban wards were listed, and the required numbers of primary sampling units for the survey were selected using probability proportion to size method. Villages or urban wards were the primary sampling units for the survey. In the selected village, the interviewers started the interview from any one side of the village, following the right-hand approach by choosing every second household until the required number is achieved. The selection of households in the village was carried out, considering at least one eligible respondent in the household. The eligible respondents were unmarried adolescents in the age group of 10–19 years residing in the area for the past 6 months. In case a household has more than one eligible respondent then, only one out of the eligible respondents was interviewed, and the selection of such respondent was made through a lottery method.

Similarly, in urban areas from each ward, we selected two colonies (the bigger one) randomly. Similar to rural areas, all other processes of selection of respondents were done in the same fashion. We applied a circular systematic random sampling method to achieve the target sample in each primary sampling unit. It needs mention here that an equal number of both male and female adolescents were selected for the study. Around 78 villages and 45 urban wards were the primary sampling units.

### Sample Size

Using a 25.7% prevalence of underweight (BMI < 18.5 kg/m^2^) among women of reproductive age (15–49 years) (6), at 95% confidence level, 5% absolute error, design effect of 1.5, and 15% dropout rate, the sample size was calculated at 534. The design effect of 1.5 was considered because of the stratification of the population into districts, blocks, and villages (arising out of stratified random sampling). A dropout rate of 15% was considered amidst the assent or consent required from adolescents and their parents (for which they do not agree at times) and refusal of adolescents to give time for the interviews as they are busy in household chores or agriculture fields or schools. This sample size of adolescents was interviewed at each of the three sites (one rural and urban site in Bihar and one rural site in Assam) and divided equally between boys and girls. The sample size calculation and equal division into girls and boys was a part of the baseline assessment of the project.

### Survey Process

A team of 10 investigators at each site identified the eligible respondents through visits to their houses after school hours. An induction training of the investigators on the questionnaire followed by telephonic mentoring on a weekly basis was done. All the interviews were conducted in the local language (Indian languages). For instance, in Bihar and Assam, Hindi and Assamese were used, respectively, for the interview. Data were collected between October and December 2017.

### Survey Instrument

Questionnaire: It consisted of questions on socio-demographic characteristics, dietary intake, history of tobacco use, and the consumption of alcohol. The socio-demographic characteristics included questions on age, gender, residence (rural or urban), social class (scheduled caste or tribe or special class or non-marginalized class), religion (Hindu, Muslim, and others), and years of schooling. Furthermore, we asked adolescents about the possession of below the poverty line card (BPL). The households identified after population-based surveys are distributed BPL cards. BPL status is based on socio-economic indicators and a minimum annual family income, which varies across states (16). In India, social class indicates a hereditary, endogenous, closed, and immutable group associated with an occupation and a particular position in the social hierarchy. Social classes such as scheduled caste or tribe or other special classes are considered marginalized classes (17). We asked adolescents if they worked outside the home for money. A structured and interviewer-based food frequency questionnaire (FFQ) of 9 food groups was used to assess the dietary intake. We adopted the pre-validated FFQ questionnaire used in the fourth round of the National Family Health Survey (NFHS-4) (6). The questions in FFQ had four possible responses, namely daily, weekly, occasionally, and never. The nine food groups included pulses (beans), milk (milk products), fruits, green, including leafy vegetables, eggs, fish, meat (chicken), snacks (fried foods), and cold drinks (other beverages). Cereals (wheat/rice/corn) were not included in the original survey, but it is known that wheat and rice consumption forms a major part of the diet of the Indian population (18). Furthermore, the questionnaire did not have any questions pertaining to the quantity or level of food consumption. Hence, portion sizes were not calculated. We employed a single FFQ method to assess the dietary patterns, which has been acknowledged to be sufficient to capture habitual dietary intake in previous studies (19). The respondents were adolescents themselves.

### Ethical Considerations

We were cognizant of the fact that matters concerning adolescents are very sensitive; therefore, before the administration of the study, ethical clearance was taken by MAMTA Ethical Review Board. The consent and assent forms were developed as per the standard guideline, and due approvals were also taken from the said Board. Before each interview, consent and assent forms were read-out by the investigator, and once agreed by both parents and adolescents, and then only the interview was conducted.

### Statistical Analysis

Data were expressed as frequency (percentages) for categorical variables. Besides, cluster analysis was performed to identify the dietary patterns by means of a 2-step procedure. This procedure grouped participants into clusters based on log-likelihood distances between observations, which assumed multinomial distribution for categorical variables. Subsequently, the clusters were treated as separate observations. Schwarz Bayesian Criterion was used to arrive at an initial number of clusters, which were 4 (Silhouette coefficient = 0.2). However, in the subsequent calculations, we pre-fixed the number of clusters based on nine food groups to be 2.

After multiple runs with different food groups in the first place and simultaneously performing a chi-square test to assess if all the food groups varied between different clusters, we decided to take only eight food groups. Pulses/beans category was removed from the final analysis as it did not vary between the two clusters (*p* > 0.05). The ratio of the larger to the smaller cluster, in the final analysis, was 3.25, and the Silhouette coefficient was 0.3. We compared clusters based on socio-demographic characteristics using binomial logistic regression for unadjusted and adjusted associations, respectively. Associations between clusters and socio-demographic characteristics were expressed as odds ratio (OR) and 95% confidence interval (CI). All the statistical analysis was performed using IBM SPSS Statistics for Windows version 25.0 (IBM Corp., Armonk, NY, USA).

## Results

A nearly equal number of adolescents belonged to early (46%) and late adolescence (54%) groups (Table 1). Around two-thirds of boys and girls were from the rural areas, as only one urban site from Munger was selected. Similarly, 65–63.5% of families of adolescents had below the poverty line card. More than two-thirds of adolescents were Hindus. Among the food groups, nearly 72% of girls and 60% of boys consumed green vegetables, including leafy vegetables, on a daily basis (Table 2). Likewise, three-fourths or more adolescent boys and girls consumed pulses/beans on a daily basis. More than two-thirds of boys and girls did not consume fruits even on a weekly basis.

**Table 1:** Percent distribution of adolescent boys and girls with respect to selected background characteristics in two districts of Bihar and Assam.

**Variable**	**Adolescent girls**	**Adolescent boys**
	**(*n* = 826) *N* (%)**	**(*n* = 811) *N* (%)**
**Age groups (years)**		
Early adolescence (10–14)	389 (47.0)	365 (45.0)
Late adolescence (15–19)	437 (53.0)	446 (55.0)
**Area**		
Rural	548 (66.3)	549 (67.7)
Urban	278 (33.7)	262 (32.3)
**Religion**		
Hindu	555 (67.2)	582 (71.8)
Muslim	266 (32.2)	228 (28.1)
Others	5 (0.6)	1 (0.1)
**Education status**		
Up to primary (1–5th grade)	150 (19.2)	190 (24.0)
Middle class (6–8th grade)	241 (30.8)	209 (26.4)
Secondary class (9 and 10th grade)	263 (33.6)	263 (33.2)
Senior secondary class and above (11th and above)	129 (16.5)	129 (16.3)
Missing	20	43
**Social class**		
Scheduled caste/tribe	290 (35.1)	230 (28.4)
Other special class	247 (29.9)	290 (35.8)
Non-marginalized class	289 (35.0)	291 (35.9)
**Possess below the poverty line card**		
Yes	536 (64.9)	515 (63.5)
No	251 (30.4)	282 (34.8)
Don't know	39 (4.7)	14 (1.7)
**Worked outside home**		
Yes	96 (11.6)	41 (5.1)
No	730 (88.4)	770 (94.9)

*SD, Standard Deviation; p-value < 0.05 was considered statistically significant*.

**Table 2:** Percent distribution of adolescents by frequency of consumption of specific food groups.

**Food groups**	**Adolescent girls**	**Adolescent boys**	***P-*value**
	**(*n* = 826) *N* (%)**	**(*n* = 811) *N* (%)**	
**Green, including leafy vegetables**			
Daily	592 (71.7)	480 (59.2)	<**0.001**
Weekly	83 (10.0)	101 (12.5)	
Sometimes	124 (15.0)	207 (25.5)	
Never	27 (3.3)	23 (2.8)	
**Fruits**			
Daily	72 (8.7)	29 (3.6)	<**0.001**
Weekly	162 (19.6)	158 (19.5)	
Sometimes	572 (69.2)	611 (75.3)	
Never	20 (2.4)	13 (1.6)	
**Egg**			
Daily	55 (6.7)	41 (5.1)	**0.002**
Weekly	218 (26.4)	213 (26.3)	
Sometimes	479 (58.0)	518 (63.9)	
Never	74 (9.0)	39 (4.8)	
**Fish**			
Daily	44 (5.3)	11 (1.4)	<**0.001**
Weekly	239 (28.9)	109 (13.4)	
Sometimes	458 (55.4)	643 (79.3)	
Never	85 (10.3)	48 (5.9)	
**Chicken/meat**			
Daily	18 (2.2)	0	<**0.001**
Weekly	207 (25.1)	80 (9.9)	
Sometimes	518 (62.7)	693 (85.5)	
Never	83 (10.0)	38 (4.7)	
**Fried foods**			
Daily	31 (3.8)	6 (0.7)	<**0.001**
Weekly	78 (9.4)	31 (3.8)	
Sometimes	478 (57.9)	636 (78.4)	
Never	239 (28.9)	138 (17.0)	
**Aerated drinks**			
Daily	3 (0.4)	0	<**0.001**
Weekly	53 (6.4)	27 (3.3)	
Sometimes	547 (66.2)	744 (91.7)	
Never	223 (27.0)	40 (4.9)	
**Milk/curd**			
Daily	242 (29.3)	258 (31.8)	<**0.001**
Weekly	91 (11.0)	247 (30.5)	
Sometimes	372 (45.0)	264 (32.6)	
Never	121 (14.6)	42 (5.2)	
**Pulses/beans**			
Daily	711 (86.1)	608 (75.0)	<**0.001**
Weekly	54 (6.5)	137 (16.9)	
Sometimes	58 (7.0)	56 (6.9)	
Never	3 (0.4)	10 (1.2)	

*p-values < 0.05 are highlighted in bold*.

**Figure 1:**
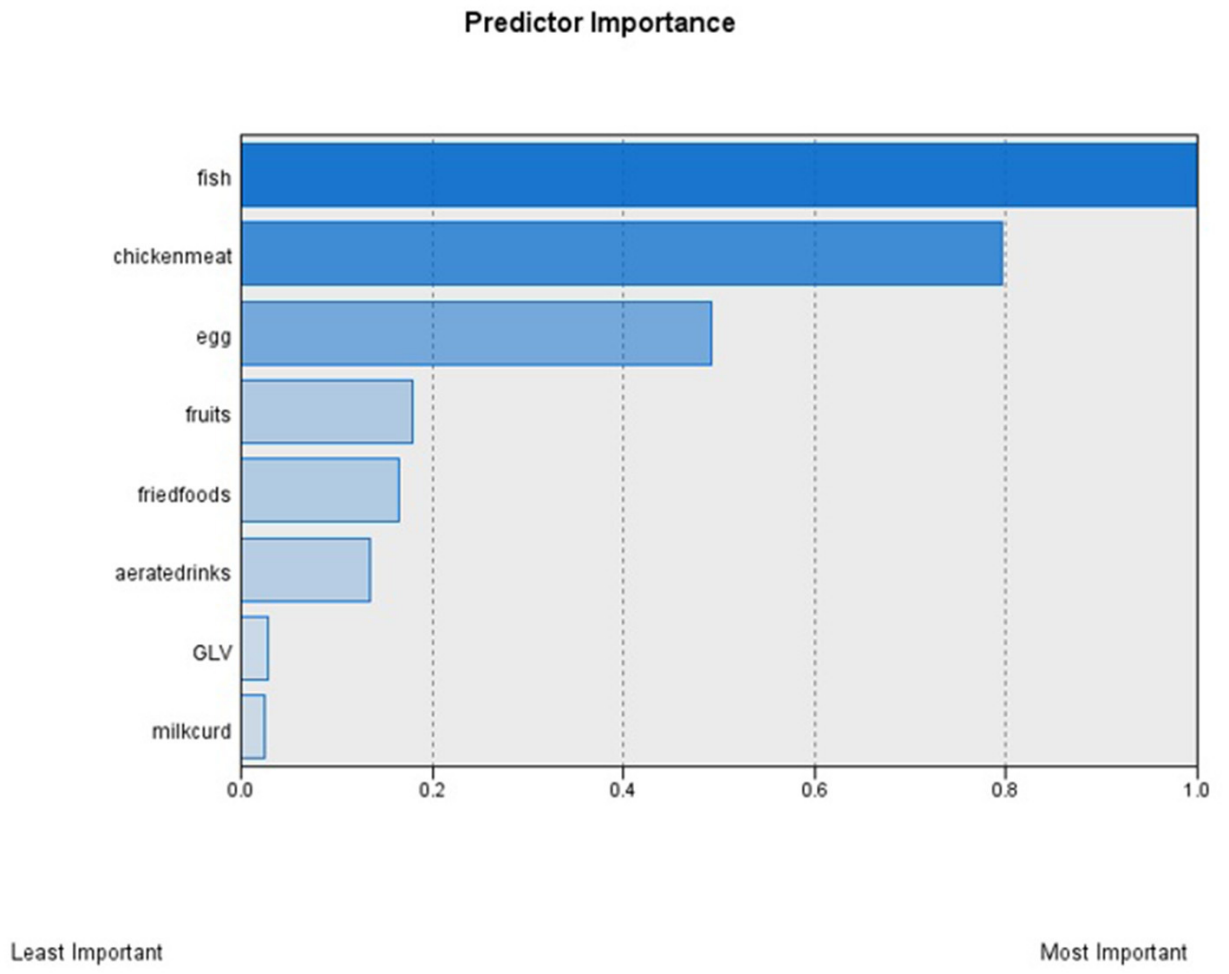
Predictor importance of the selected food groups (*n* = 8). GLV, Green, including leafy vegetables. Contribution of each food item to the clustering solution as reported from the Two-Step cluster analysis. This shows the index of relative importance of each food item as identified by the Two-Step cluster analysis. Fish and chicken/meat had high predictor importance, meaning that they are responsible for the wide difference between the clusters. On the contrary. Green leafy vegetables (GLV) and milk/curd had lowest predictor importance. So, they are important in predicting a model.

We found two clusters in the analysis (Table 3). Fish and chicken/meat had the highest predictor importance in the two-step cluster analysis (Figure 1), which means that they are responsible for the wide difference between the clusters and are important predictors in estimating the model. The size of the larger cluster was 1,252 (76.5%), and the smallest cluster was 385 (23.5%). The first cluster had a higher percentage of adolescents consuming green vegetables, including leafy vegetables on a daily basis (68.8 vs. 54.5% in the second cluster), and fruits and fried foods (1.6–1.7 times the second cluster) sometimes than the second cluster. On the contrary, the second cluster had a large number of adolescents consuming meat/chicken, eggs and fishes on a weekly basis (65–80%) and milk/curd on a daily basis (~40%) compared to the first cluster. Hence, we named the first cluster as “low-mixed diet” and the second cluster as “high-mixed diet.” Girls had a higher consumption of fish and chicken/meat compared to boys and also, these two food items had the highest predictor importance in the cluster analysis. Other food items such as milk and curd or green leafy vegetable had similar consumption between the two genders.

**Table 3:** Percent distribution of two clusters according to selected food groups.

**Food groups**	**Cluster-1 (Low-mixed diet)**	**Cluster-2 (High-mixed diet)**
	**Frequency (%)**	**Frequency (%)**
Green, including leafy vegetables	Daily (68.8)	Daily (54.5)
Fruits	Sometimes (80.3)	Sometimes (46.2)
Egg	Sometimes (75.2)	Weekly (65.5)
Fish	Sometimes (86.3)	Weekly (81.3)
Chicken/meat	Sometimes (88.7)	Weekly (69.6)
Fried foods	Sometimes (74.4)	Sometimes (47.3)
Aerated drinks	Sometimes (84.3)	Sometimes (61.0)
Milk/curd	Sometimes (40.2)	Daily (39.7)

Adolescent boys had 3.6 times higher odds of consuming a low-mixed diet compared to girls (Table 4). Adolescents who were illiterate or had obtained education up to primary or middle grades had higher odds of having a low-mixed diet as compared to adolescents who obtained education up to senior secondary grade and above. Hindus had higher odds of low-mixed diet than Muslims in the unadjusted analysis, but not so after adjustments for socio-economic parameters. Social marginalization, such as belonging to scheduled caste or scheduled tribe and other special classes, was a significant predictor of the use of a low-mixed diet.

**Table 4:** Logistic regression analysis showing results of unadjusted and adjusted odds ratio of the likelihood of the low-mixed diet with respect to selected background characteristics.

**Variables**	**Unadjusted odds**	***p-*value**	**Adjusted odds**	***p-*value**
	ratio (95% CI)^*^		ratio (95% CI)^*^	
**Age groups (years)**				
10–14	1.09 (0.86–1.37)	0.459	0.88 (0.63–1.23)	0.468
15–19	*Reference*		*Reference*	
**Gender**				
Male	3.44 (2.68–4.43)	**<0.001**	3.63 (2.79–4.73)	**<0.001**
Female	*Reference*		*Reference*	
**Area**				
Rural	0.79 (0.61–1.01)	0.063	0.84 (0.64–1.11)	0.239
Urban	*Reference*		*Reference*	
**Religion**				
Hindu	1.89 (1.49–2.40)	**<0.001**	1.17 (0.81–1.69)	0.404
Others	2.35 (0.27–20.29)	0.437	2.65 (0.28–25.25)	0.397
Muslim	*Reference*		*Reference*	
**Education status**				
Up to primary (1–5th grade)	1.60 (1.09–2.35)	**0.015**	2.15 (1.32–3.52)	**0.002**
Middle class (6–8th grade)	1.39 (0.98–1.98)	0.067	1.92 (1.22–3.00)	**0.004**
Secondary class (9 and 10th grade)	1.12 (0.80–1.56)	0.504	1.27 (0.88–1.83)	0.190
Senior secondary class and above (11th and above)	*Reference*		*Reference*	
**Social class**				
Scheduled caste/tribe	1.83 (1.39–2.41)	**<0.001**	1.94 (1.30–2.87)	**0.001**
Other special class	2.33 (1.75–3.10)	**<0.001**	2.26 (1.51–3.39)	**<0.001**
Non-marginalized class	*Reference*		*Reference*	
**Possess below poverty line card**				
Yes	1.04 (0.81–1.32)	0.757	1.10 (0.84–1.43)	0.481
Don't know	1.56 (0.74–3.28)	0.241	2.27 (1.01–5.07)	0.046
No	*Reference*		*Reference*	
**Work outside**				
Yes	1.01 (0.67–1.52)	0.963	1.33 (0.85–2.10)	0.208
No	*Reference*		*Reference*	

*CI, Confidence interval; p-value < 0.05 was considered statistically significant*.

* *Cluster 2 was the reference category. p-values < 0.05 are highlighted in bold*.

## Discussion

In the present study, we found two major dietary patterns, namely a low- and high-mixed diet. The low-mixed diet had daily consumption of green vegetables, including leafy vegetables, with less frequent consumption of other foods. On the other hand, the high-mixed diet had more frequent consumption of chicken/meat, egg, and milk/curd apart from green vegetables. Adolescent boys, and adolescents with lower grades of education, and from marginalized classes had higher odds of low-mixed diet compared to their respective counterparts.

The findings of the present study indicated daily consumption of green vegetables, including leafy vegetables and pulses, among more than 60% of adolescents. Similar findings with regard to the consumption of vegetables (both green leafy and other vegetables) among adolescents were reported in another study (20). The NFHS-4 (2015-16) reported at least once a week consumption of both vegetables and pulses among 83–89% of adolescent boys and girls (15–19 years) (6). Another study reported daily consumption of vegetables by only one-third of adolescents (21). The plausible explanation for the frequent consumption of green vegetables in our study could be a single category for all types of green vegetables compared to separate categories in other studies. Furthermore, most of the population in our study (nearly two-thirds) belonged to the rural areas that had easy access to and availability of locally-grown vegetables compared to urban areas. The consumption of fruits was poor in our study. Only one-fifth of adolescents consumed fruits on a weekly basis. Likewise, other studies from Uttar Pradesh and Kolkata reported low consumption of fruits among school-going adolescents (20, 22).

In the present study, the majority of the adolescents consumed egg, fish, or meat sometimes, and 5–10% of adolescents never consumed them. Low consumption of non-vegetarian food items could be partly explained by the cost factor (high cost of non-vegetarian food items) and easy availability of vegetables in villages (67–71%). Furthermore, it has been highlighted in the previous studies that the majority of Indians have a vegetarian dietary pattern influenced by religious beliefs (13, 23). It is surprising to see a low frequency of consumption of milk or milk products during adolescence in our study, which is a period of growth and development and requires a high intake of calcium and proteins. But, unfortunately, previous literature noted similar findings (20, 24). The study based by the National Sample Survey Organization (NSSO) reported that the consumption of milk has increased marginally or reduced in states like Bihar and Assam in the last 25 years (25).

The two clusters defined in the present study have been reported previously in another study using a similar FFQ questionnaire (26). The low-mixed diet was the predominant dietary pattern among adolescents in our study (76.5%). Amongst adolescents in the low-mixed cluster, quite large proportions (68.8%) reported daily consumption of green vegetables, including leafy vegetables, and the consumption of fried foods and aerated drinks (74–84%) sometimes. This may suggest two different possible conditions. Firstly, the high cost of non-vegetarian items, such as fish and meat, and easy availability of green leafy vegetables in the villages. Secondly, it could be because of the lack of knowledge about a balanced diet and healthy foods, including sources of protein or calcium like milk or curd. Furthermore, fish/meat/egg had the highest contribution in the clustering solution because they are less often eaten by the population in Bihar compared to other states (23, 27, 28). A similar study among rural adolescents and adults informed of lack of food diversity with less frequent consumption of fruits, and non-vegetarian food items in India, leading to nutritional deficiencies and inadequacy (29).

Globally, studies have documented a healthier dietary pattern among females. Multiple assumptions have been stipulated for the gender differences in dietary patterns, including weight-control behaviors and greater health consciousness among girls and masculinity ideation discouraging men from eating a healthy diet (20, 29). However, it will be difficult to conclude in our study that boys had a lower probability of eating a healthy diet compared to girls as both boys and girls consume veggies frequently, girls consume meats and fish more frequently, and both groups consume fried foods and aerated drinks infrequently. Increased access to fish/chicken/meat by girls should not be seen as a proxy for healthier diet consumption; instead, the underlying dynamics of intrahousehold food distribution need to be understood better and figured out why such differentials prevail.

The higher odds of consuming the low-mixed diet among less educated and marginalized populations suggest socio-economic inequalities in food consumption among the Indian population. Our findings are congruent with other studies reporting similar poor intake of nutrition among socially and economically backward populations (30, 31). Similar to our results, education has been found to influence the intake of a variety of food groups more than the economic status in other studies (32, 33). This highlights the need to ensure food security by improving the availability, access, and utilization of food to poor and marginalized populations (34). The targeted public distribution system in India bridged this gap by providing nutrition supplements to poor people. However, multiple challenges, including inaccurate identification of households, leakages, and diversions of food grains, inadequate storage capacity, and poor quality of food grains, exist in the targeted public distribution system (35).

Furthermore, it is acknowledged that regular family meals may promote the uptake and maintenance of healthy eating behaviors among adolescents. Regular family-oriented meals will improve the intake of fruits, vegetables, dietary fiber, and vitamins and cut down the intakes of saturated and trans fats (36).

## Limitations

The study results should be interpreted with caution due to the following limitations. First, we could not include other food groups like roots and tubers, nuts or seeds, cereals, sweets, and milk-based beverages in the analysis due to the lack of questions on each of these groups in the validated NFHS-4 questionnaire. The inclusion of these food groups might have resulted in more distinct clusters. Second, although a single FFQ method is good for exploring the dietary patterns, there could be a recall bias of remembering the consumption of food items over weeks or months. Third, we could not obtain the dietary patterns of adults and reflect how different adolescents are from adults in the same families. Lastly, we could not collect data regarding the intake of cereals in the dietary intakes and quantify energy intakes. This would have explained the gender differences seen in the study.

## Conclusions

In this study, we observed two dietary patterns, low- and high-mixed diets, among adolescents. The low-mixed diet, common among 76% of adolescents, had a low intake of non-vegetarian food items and milk. Adolescent boys and adolescents with lower education status and from marginalized classes had higher odds of consuming a low-mixed diet compared to their counterparts. Although Hindus had higher odds of low-mixed diet in the unadjusted model, the association became insignificant in the adjustment model.

The low-mixed diet with less frequent consumption of non-vegetarian food and milk can lead to micronutrient deficiencies and undernutrition among adolescents. Socio-cultural influences on the dietary intakes of Indian populations are significant and inevitable. Taking cognizance of these findings, public health interventions should target behavior change communication that aims to increase dietary diversity along with the intake of nutrients. This should involve a comprehensive health promotion strategy of educating adolescents and their parents/teachers on nutrition, besides strengthening nutrition supplementation programs of the government, such as mid-day meals and weekly iron-folic acid supplementation. Furthermore, food supplementation programs can ensure the provision of costly protein-rich food items (non-vegetarian) and milk through *Anganwadi* centers or mid-day meals in schools. Since adolescent boys had a higher consumption of the low-mixed diet, the reasons of the same should be explored, and the gaps need to be addressed.

## Data Availability Statement

The raw data supporting the conclusions of this article will be made available by the authors, without undue reservation.

## Ethics Statement

The studies involving human participants were reviewed and approved by MAMTA Ethical Review Board. Written informed consent to participate in this study was provided by the participants' legal guardian/next of kin.

## Author Contributions

SMa and JK contributed to the conceptualization and design of the study, training of the field investigators on the questionnaire, monitoring the data collection, and reviewing or editing of the final manuscript. SS led the statistical analysis, interpretation of the data, and wrote the draft manuscript. SMe contributed to the conceptualization of the study and reviewed and edited the manuscript. C was involved in statistical analysis and editing of the final manuscript. All authors contributed to the article and approved the submitted version.

## Funding

This study was a part of implementation science project funded by ITC Company under Corporate Social Responsibility. However, the funder was not involved in any component of the study, including designing of the questionnaire, data collection, analysis, or writing the paper.

## Conflict of Interest

The authors declare that the research was conducted in the absence of any commercial or financial relationships that could be construed as a potential conflict of interest.

## Publisher's Note

All claims expressed in this article are solely those of the authors and do not necessarily represent those of their affiliated organizations, or those of the publisher, the editors and the reviewers. Any product that may be evaluated in this article, or claim that may be made by its manufacturer, is not guaranteed or endorsed by the publisher.
